# First record of the genus *Prosopistoma* Latreille, 1833 (Ephemeroptera, Prosopistomatidae) in Taiwan

**DOI:** 10.3897/zookeys.473.8787

**Published:** 2015-01-20

**Authors:** Rita S. W. Yam

**Affiliations:** 1 Department of Bioenvironmental Systems Engineering, National Taiwan University, Taipei 10617, Taiwan R.O.C.

**Keywords:** Mayfly, Prosopistomatidae, new record, nymph, morphology, Taiwan

## Abstract

The finding of three immature nymphs of *Prosopistoma* Latreille, 1833 (Ephemeroptera, Prosopistomatidae) in an upstream site of Baishih River represents the first record of this rarely collected genus in Taiwan. These nymphs were discovered through extensive monthly sampling at the riffle habitats from 13 undisturbed sites over two years (Dec 2008–Nov 2010). The coloration pattern of the collected immature nymphs in Taiwan is similar to the immature stage of *Prosopistoma ocellatum* and *Prosopistoma annamense*, two species which have been found in similarly undisturbed, upland forested-stream habitats.

## Introduction

The Prosopistomatidae is a monogeneric family of Ephemeroptera, and is considered as rarely collected. At present, more than 20 known species of *Prosopistoma* Latreille, 1833 have been described from the Palaearctic, Oriental, Australasian, and Afrotropical regions (see review by Barber-James 2009, Shi and Tong 2013). The Oriental region (12 species) represents the most species-rich area for this genus (Lieftinck 1932, Peters 1967, Liu et al. 1984, Soldán and Braasch 1984, Tong and Dudgeon 2000, Sartori and Gattolliat 2003, Zhou and Zheng 2004, Barber-James et al. 2008, Barber-James 2009, Shi and Tong 2013). No *Prosopistoma* has been mentioned in Taiwan despite records of this genus in the nearby continental China and other major Asian Pacific islands (e.g. Philippines, Borneo, Java and Sumatra).

Studies on the diversity of mayfly in Taiwan started from Ulmer (1912) who mentioned nine species with decription of four new species of *Ephemera*, *Isonychia* and *Ecdyonurus*. The taxonomic records of mayfly extensively increased in 1990’s (e.g. Kang and Yang 1994a, b, c, d, e, 1995, 1996a, b, Kang et al. 1994, Bae 1997) when 45 species of Ameletidae, Baetidae, Heptageniidae, Leptophlebiidae and Caenidae were described from more than 100 localities (see review in Soldán and Yang 2003). To date, at least 65 species (28 genera and 9 families) of mayflies are recorded in Taiwan (Soldán and Yang 2003). However, no *Prosopistoma* has been discovered in Taiwan in the last >100 years of entomological studies, suggesting its rarity in Taiwan. The present study is to report the first record of *Prosopistoma* in Taiwan and consequently its geographic extension in the Oriental region.

## Materials and methods

**Figure 1.**
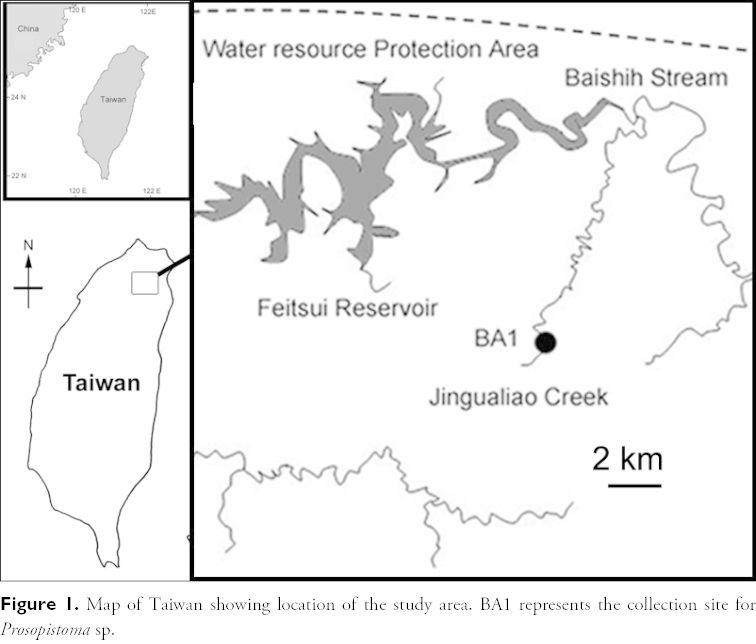
Map of Taiwan showing location of the study area. BA1 represents the collection site for *Prosopistoma* sp.

**Figure 2.**
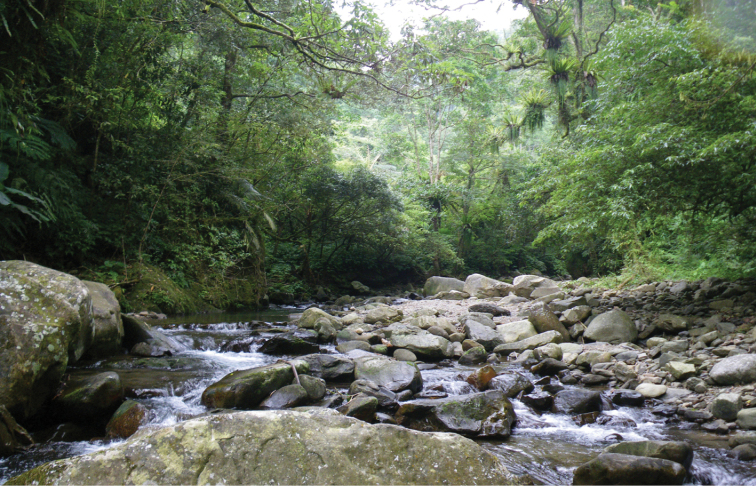
Photograph of the collection site BA1.

Extensive monthly surveys for benthic macroinvertebrates at the riffle habitats from 13 undisturbed, upland sites of the Baishih River from the Water Resource Protection Area of the Feitsui Reservoir in New Taipei City, Taiwan were conducted for two years from Dec 2008 to Nov 2010 (Fig. 1). *Prosopistoma* nymphs were only discovered at the study site BA1 (Fig. 2), and samples were collected using hand nets from the stony streambed. All materials were collected by the author, and preserved in 95% ethanol. The specimens were examined and dissected under stereomicroscopes. The dissected mouthparts and legs were investigated using a compound light microscope. One specimens were air-dried, gold coated and examined using a Scanning Electron Microscope. All specimens are kept in the Ecology and Conservation Laboratory, Department of Bioenvironmental Systems Engineering, National Taiwan University, Taiwan (ECL). Terminology follows Kluge (2004) and Barber-James (2010).

## Taxonomy

### Family Prosopistomatidae Lameere, 1917 Genus *Prosopistoma* Latreille, 1833

#### Prosopistoma sp.

Taxon classificationAnimaliaEphemeropteraProsopistomatidae

Figs 3 , 4 , 5 , 6

##### Material examined.

ECL-20100701-1: 1 nymph, TAIWAN, Baishih River (24.882695°N, 121.656242°E), 1.vii.2010. ECL-20100707-2: 1 nymph, TAIWAN, Baishih River (24.882695°N, 121.656242°E), 7.vii.2010. ECL-20100707-3: same data as ECL-20100707-2.

##### Description.

**Figure 3.**
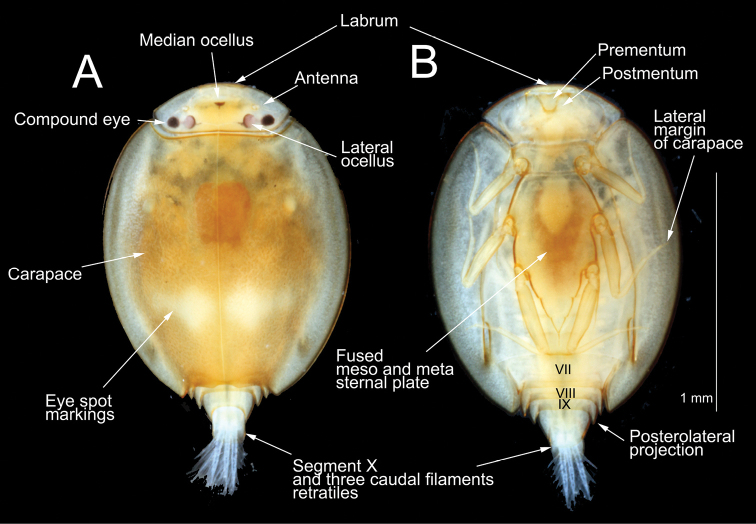
*Prosopistoma* sp. whole nymph: **A** Dorsal view **B** Ventral view.

Immature nymph. Body length 1.5–2 mm, excluding caudal filaments. Head yellowish with a small red median ocellus, width about 3 times longer than length. Carapace coloration orange, with two white eye-spot markings on each side close to the mid line, about 2/3 of the distance from the base of the head. Distal end of carapace with a concave exhalent notch (Fig. 3A–B).

**Figure 4.**
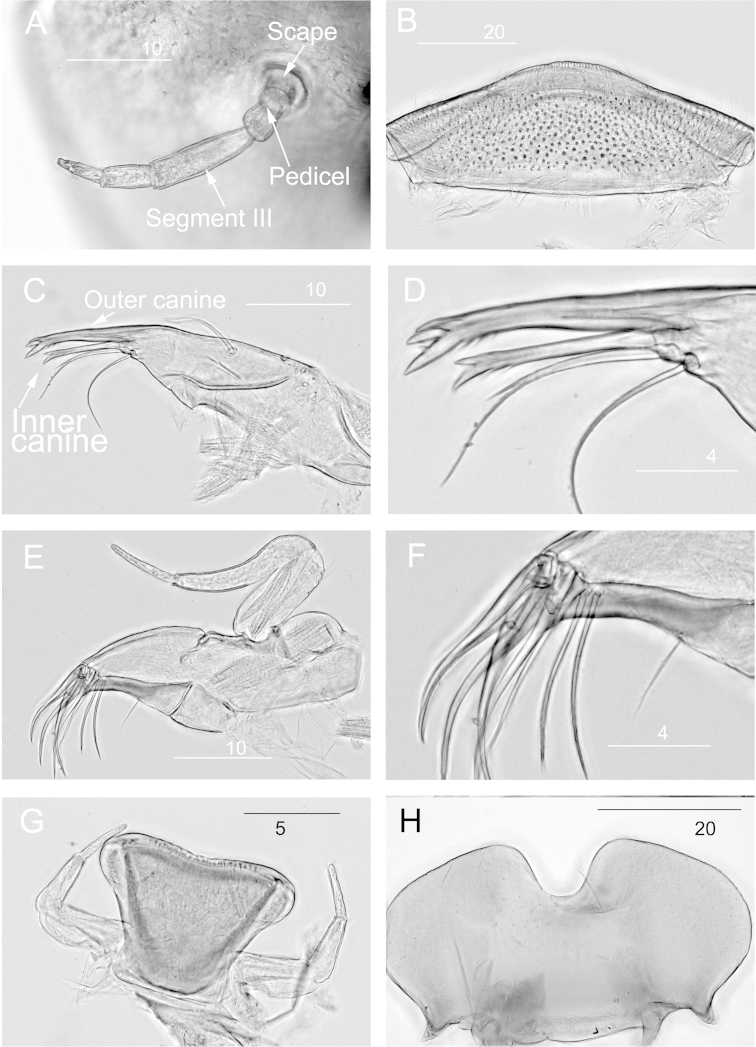
*Prosopistoma* sp.: **A** Antenna **B** Labrum **C** Mandible **D** Magnified view of inner and outer canine of mandible **E** Maxillae **F** Tip of Maxillae **G** Prementum with labial palps **H** postmentum. Scale bar in μm.

**Head.** Antenna with 5 segments, segment III longest (Fig. 4A). Labrum narrow, 3 times wider than long, surface with stout setae, anterior margin with sparse setae (Fig. 4B). Left and right mandibles similar, outer canine longer than the inner one, outer canine with three apical teeth, outer tooth the smallest with smooth outer margin, inner tooth the largest, with three short spines along the inner margin. Inner canine with two apical teeth, inner tooth larger with outer margin smooth, inner margin with two small spines. Two smooth setae below the inner tooth (Figs 4C–D). A single simple seta present lateromedially on each mandible (Fig. 4C). Maxillae with1 rigid canine at tip, with 3 subequal dentisetae and 3 stout setae (Fig. 4E–F). A simple seta at 2/3 of the sclerotized section of galea-lacinia (Fig. 4E-F). Maxillary palp 3-segmented, with segment II the longest (Fig. 4E). Labium composed of prementum and postmentum. Prementum trapezoid, cutting edge with fine teeth (Fig. 4G). Postmentum with large notch, to house the prementum (Fig. 4H). Labial palp 3-segmented, with the second the longest (Fig. 4G).

**Figure 5.**
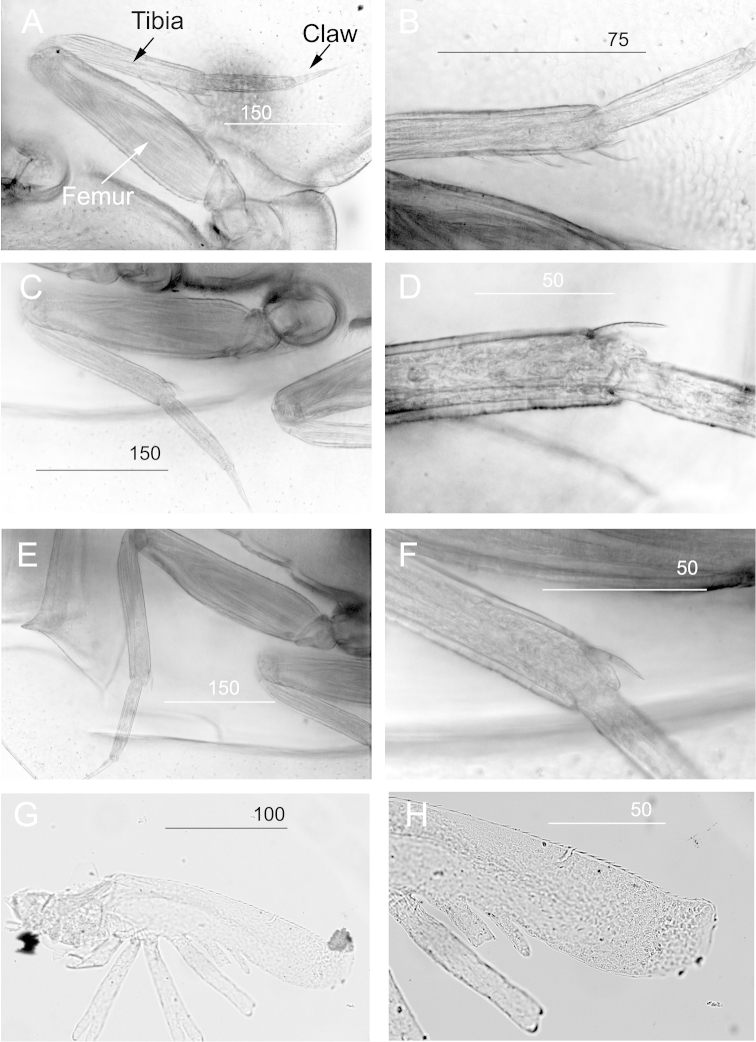
*Prosopistoma* sp.: **A** Leg I **B** Apex of ventral margin of tibia showing 4 serrated spines **C** Leg II **D** Apex of ventral margin of tibia of leg II **E** Leg III **F** Apex of ventral margin of tibia of leg III **G** Gill I **H** Upper lamellae portion of gill I. Scale bar in μm.

**Figure 6.**
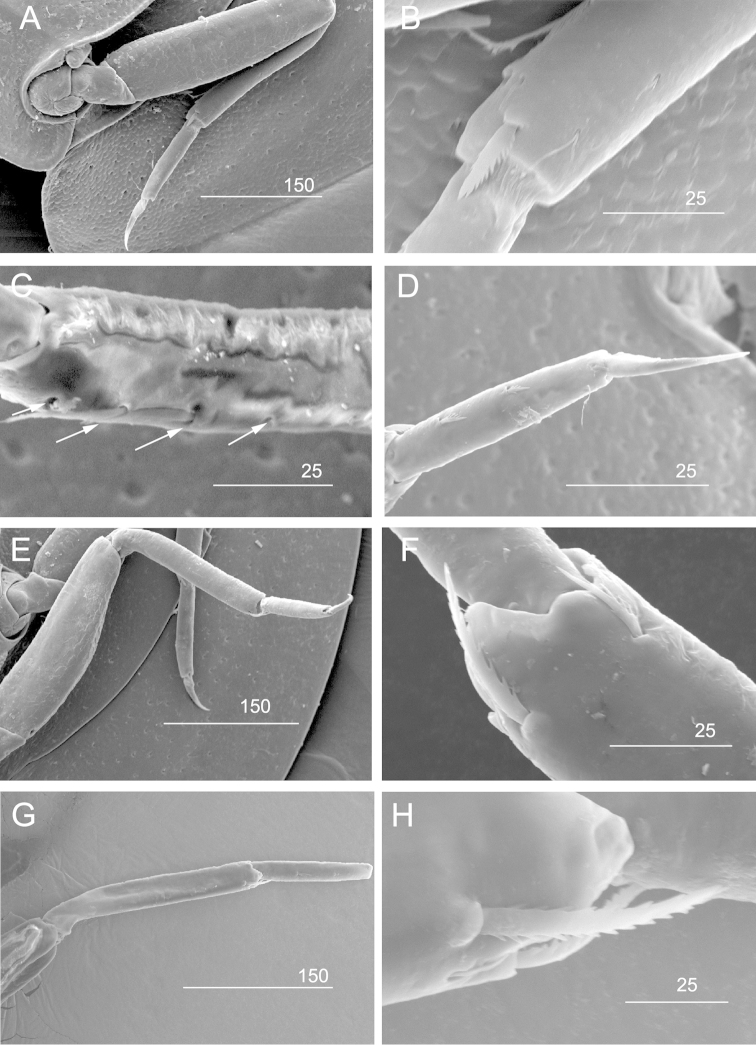
SEM of *Prosopistoma* sp.: **A** Leg I **B** Dorsal margin of tibia of leg I **C** Ventral margin of tibia of leg I, showing 4 serrated spines (indicated by white arrows). Note spines 1 and 4 are broken **D** Claw of leg I **E** Leg II **F** Fore-tibia of leg II **G** Leg III **H** Apex of fore-tibia of leg III. Scale bar in μm.

**Legs.** Dorsal and ventral margins of fore femur smooth (Figs 5A, 6A). Ventral margin of fore tibia with a row of 4 serrated setae (Figs 5B, 6C). Apical serrated setae on tibiae of legs II and III (Figs 5D, F, 6B, E–H). Claws of all legs sharp and without denticles (Fig. 6D).

**Abdomen.** Posterolateral projections of abdominal segments VII-IX sharp and with pointed apex (Fig. 3B). Three caudal filaments short and setose (Fig. 3B). Gill I with long upper lamellate portions, lamellate margin serrated, lower portions divided into several branches (Fig. 5G–H). Gill II leaf-like unbranched. Gill VI tiny, unbranched.

**Distribution.** At present, this unnamed species is only recorded in Baishih River from Taiwan.

**Habitat.** The collection site BA1 is an undisturbed forested-stream (356 m a.s.l., Fig. 2) with wetted width (6.3–10.5 m) and depth (0.2–0.7 m) relatively constant throughout the year. This site is generally oligotrophic (nitrate-nitrogen < 0.01 mg/L, ammonium-nitrogen = 1.40±0.28 mg/L, total phosphorus = 0.10±0.06 mg/L). Nymphs were found within the riffles with accumulated leaf packs on the bed substrates dominated by gravels and pebbles, moderate to high current velocity (26.7–65.1 cm/s) and high dissolved oxygen level (7.3–9.6 mg/L). Nymphs were rare and they contribute to the relative composition of the mayfly community by 0.19% during the study period. Dominant families of mayfly nymphs collected in the same habitat included Baetidae, Heptageniidae, Leptophlebiidae and Caenidae.

##### Remarks.

According to the diagnostic key in Shi and Tong (2013), the immature nymphs of *Prosopistoma* sp. are morphologically similar to *Prosopistoma ocellatum*. The coloration pattern of the collected immature nymphs in Taiwan is similar to the immature stage of *Prosopistoma ocellatum* and *Prosopistoma annamense*. However, as the important diagnostic characteristics, such as number of setae on fore tibia and number of antennal segments, are likely to change with ontogenetic shift, we cannot properly diagnose our specimens due to the lack of mature nymphs collected through extensive sampling in the present study.

Habitat of the nymphs of *Prosopistoma* sp. are similar to most *Prosopistoma* such as *Prosopistoma annamense*, *Prosopistoma olympus* and *Prosopistoma ocellatum*. Their habitats are generally located in the undisturbed upstream site (altitude = 200–800 m a.s.l.) commonly characterized by stony streambed, shallow water depth, and moderate to high current velocity (Soldán and Braasch 1984, Sartori and Gattolliat 2003, Shi and Tong 2013) except that nymphs of *Prosopistoma annamense* were recorded in the large urban river Xiangjiang from China (Liu et al. 1984).

In this study, the finding of three immature nymphs of *Prosopistoma* sp. from the upstream site of Baishih River represents the first record of this rarely collected genus in Taiwan. Thus, further collections should be conducted at more river sites to obtain the mature nymphs to ascertain the taxonomic status of this *Prosopistoma* sp. in Taiwan.

## Supplementary Material

XML Treatment for Prosopistoma sp.
